# Splice Variants of the Castor *WRI1* Gene Upregulate Fatty Acid and Oil Biosynthesis When Expressed in Tobacco Leaves

**DOI:** 10.3390/ijms19010146

**Published:** 2018-01-05

**Authors:** Xia-Jie Ji, Xue Mao, Qing-Ting Hao, Bao-Ling Liu, Jin-Ai Xue, Run-Zhi Li

**Affiliations:** Institute of Molecular Agriculture and Bioenergy, Shanxi Agricultural University, Taigu 030801, China; jixiajie@stu.sxau.edu.cn (X.-J.J.); maoxue@sxau.edu.cn (X.M.); haoqingting@stu.sxau.edu.cn (Q.-T.H.); liubaoling@stu.sxau.edu.cn (B.-L.L.); xuejinai@sxau.edu.cn (J.-A.X.)

**Keywords:** RcWRI1, alternative splice form, fatty acid and oil biosynthesis, castor (*Ricinus communis* L.), tobacco (*Nicotiana benthamiana* L.)

## Abstract

The plant-specific WRINKLED1 (WRI1) is a member of the AP2/EREBP class of transcription factors that positively regulate oil biosynthesis in plant tissues. Limited information is available for the role of WRI1 in oil biosynthesis in castor bean (*Ricinus connunis* L.), an important industrial oil crop. Here, we report the identification of two alternatively spliced transcripts of *RcWRI1*, designated as *RcWRI1-A* and *RcWRI1-B*. The open reading frames of *RcWRI1-A* (1341 bp) and *RcWRI1-B* (1332 bp) differ by a stretch of 9 bp, such that the predicted RcWRI1-B lacks the three amino acid residues “VYL” that are present in RcWRI1-A. The *RcWRI1-A* transcript is present in flowers, leaves, pericarps and developing seeds, while the *RcWRI1-B* mRNA is only detectable in developing seeds. When the two isoforms were individually introduced into an *Arabidopsis* *wri1-1* loss-of-function mutant, total fatty acid content was almost restored to the wild-type level, and the percentage of the wrinkled seeds was largely reduced in the transgenic lines relative to the *wri1-1* mutant line. Transient expression of each *RcWRI1* splice isoform in *N. benthamiana* leaves upregulated the expression of the WRI1 target genes, and consequently increased the oil content by 4.3–4.9 fold when compared with the controls, and *RcWRI1-B* appeared to be more active than *RcWRI1-A*. Both *RcWRI1-A* and *RcWRI1-B* can be used as a key transcriptional regulator to enhance fatty acid and oil biosynthesis in leafy biomass.

## 1. Introduction

Vegetable oils stored in plant seeds are predominantly composed of triacylglycerols (TAGs), glycerol esters with three fatty acids that serve as an energy reserve for catabolism during germination [1]. In addition to use as human food, plant oils are also important as renewable feedstocks for biofuel production, which could potentially decrease our dependence on fossil oil [2,3]. As such, developing high-yielding oil crops and creating new oil production platforms are needed to ensure sustainable supply of global vegetable oils. Understanding the molecular and biochemical mechanisms underlying fatty acid (FA)/oil biosynthesis and regulation is important to undertake this endeavor.

Current knowledge of the FA and TAG biosynthesis has been mainly obtained from the study of the model plant *Arabidopsis* [4,5]. These studies have revealed a series of enzymes that are involved in converting photosynthate sucrose to TAG [6,7,8]. The biosynthetic steps are spatially separated between different organelles and regulated by various biochemical mechanisms [9]. In particular, sucrose is converted to pyruvate (Pyr) via cytosolic or plastidic glycolytic pathways, which provides Pyr as the precursor for the acetyl-coenzyme A (CoA) molecules destined to FA synthesis in plastids. FAs with different carbon-chain length are subsequently transported out from the plastid to the cytoplasm as acyl-CoAs. At endoplasmic reticulum (ER), acyl-CoAs are used to acylate glycerol-3-phosphate (G3P) backbone, either by Kennedy pathway to produce TAGs or by acyl exchange between lipids [10,11]. The resulting TAGs within the ER membrane are then budded off as specialized structures called oil bodies surrounded by a single-layer phospholipids and proteins.

WRINKLED1 (WRI1), a member of the APETALA2/ethylene-responsive element binding protein (AP2/EREBP) transcriptional factor family [12], has been identified as a master regulator in the control of oil biosynthesis. Loss-of-function mutants of *WRI1* exhibited wrinkled and incompletely filled seeds with an 80% reduction of total TAGs when compared with the wild-type seeds [13]. WRI1 coordinates expression of the gene cluster essential in FA biosynthesis, including plastid pyruvate kinase beta subunit 1 (*PKp-β1*), pyruvate dehydrogenase E1 component alpha subunit (*PDH-E1α*), biotin carboxyl carrier protein isoform 2 (*BCCP2*), acetyl-CoA carboxylase (*ACCase*), sucrose synthase 2 (*SUS2*), and 3-ketoacyl-acyl carrier protein synthase 1 (*KAS1*) [14,15,16,17]. The promoter regions of these genes contain the AW-box (CnTnG(n)7CG) or 15-bp element (CAAAAG(T/G)AGG(G/A)APTT) which serve as the WRI1-binding sites [18]. 

WRI1 was first discovered in *Arabidopsis* [12], and its orthologs have been identified from oil seed crops such as rapeseed (*Brassica napus* L.) [19], corn (*Zea mays* L.) [20], and *Camelina sativa* [21], as well as from plants rich in oil in non-seed tissues such as oil palm (*Elaeis guineensis Jacq*) [22,23], poplar (*Populus trichocarpa* L.) [24], *Brachypo diumdistachyon* [25], and *Cyperu sesculentus* [26]. Overexpression of these diverse *WRI1* genes has led to significant increases in oil accumulation [21,23,25,26], providing a new tool for developing high-yielding oilseeds and oil-enriched non-seed biomass. 

Recently, several homologous genes of *AtWRI1* (e.g., *AtWRI2*, *AtWRI3* and *AtWRI4*) were also characterized; these genes show a similar function as *AtWRI1* although their expression patterns were different [16,23]. Moreover, three alternative splice forms (At3g54320.1-3) are predicted for *AtWRI1* and only splice form 3 (At3g54320.3) is present in multiple *Arabidopsis* tissues [23]. It is currently unknown whether all the three *AtWRI1* splice variants play roles in plant fatty acid biosynthesis. 

Castor bean (*Ricinus connunis* L.) is an important industrial oil crop rich in ricinoleic acid (90% of total oil) [27,28]. However, regulatory networks underlying oil biosynthesis and accumulation have not yet been well understood in this species [29]. In this study, we identified two alternative splice variants for the castor bean *WRI1* gene (*RcWRI1*), namely *RcWRI1-A* and *RcWRI1-B*. The two transcript isoforms displayed differential expression patterns in various castor tissues. Expression of either *RcWRI1-A* or *RcWRI1-B* increased seed oil accumulation in the *Arabidopsis wri1-1* mutant. Furthermore, transient expression of individual *RcWRI1* isoforms in *Nicotiana benthamiana* leaves significantly activated the *WRI1* target gene expressions, leading to a great enhancement of oil/TAG accumulation in leaf tissues, with *RcWRI1-B* being more active than *RcWRI1-A*. Our data provide novel insights into the WRI1-mediated regulatory networks responsible for FA biosynthesis in plant, and suggest that both *RcWRI1-A* and *RcWRI1-B* could be potentially used for engineering high-oil production crops. 

## 2. Results

### 2.1. Molecular Characterization of Castor RcWRI1

**Figure 1:**
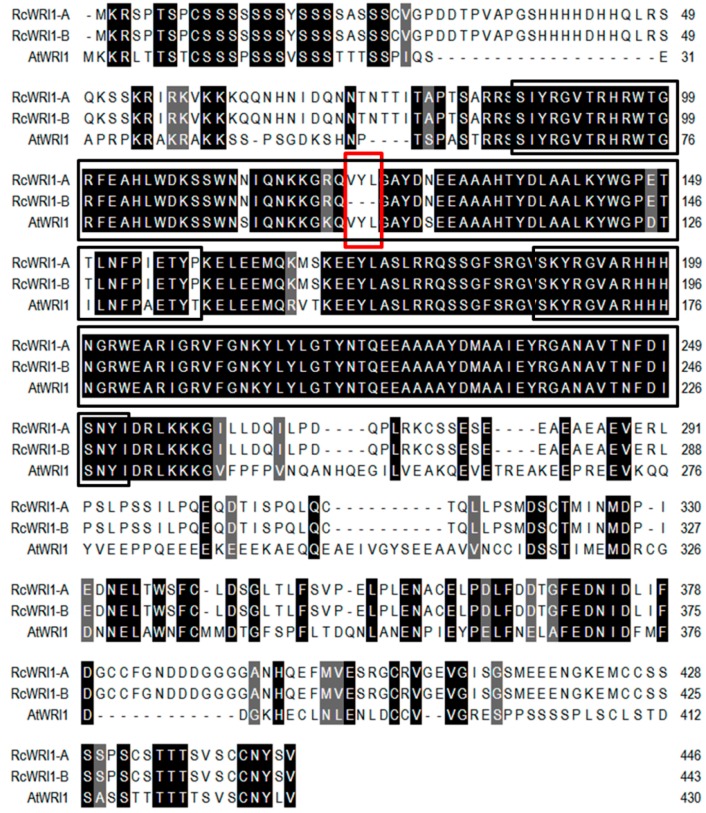
Sequence alignment of two RcWRI1 and AtWRI1 protein isoforms. The alignment was performed by the ClustalW program. Most of the difference between protein sequences of RcWRI1s and AtWRI1 occurs at the C-terminal half of the protein. “VYL” motif in AtWRI1 and RcWRI1-A is absent in RcWRI1-B. The high and low consensus amino acid residues are denoted by black and gray colors, respectively. Two AP2 domains are marked by black boxes. The sequence “VYL” present in the first AP2 domain are marked by red box.

To identify the WRI1 ortholog in castor bean, a BLASTP analysis with an e-value threshold of 1.0 × 10^−6^ was performed against the castor bean genome database using the protein sequence of AtWRI1 as a query. The highest score was obtained for the gene (LOC8283400), which we tentatively termed RcWRI1. A primer pair was designed based on the open reading frame (ORF) of this gene, which was subsequently used to amplify the cDNA encoding *RcWRI1* by RT-PCR. This experiment identified two *RcWRI1* cDNAs with different length. Sequence analysis revealed that the long form (1341 bp) of *RcWRI1* was nearly identical with the short form (1332 bp) of *RcWRI1*, with the short form lacking a stretch of 9-bp nucleotides encoding “VYL” (Figure 1). The long and short cDNAs of *RcWRI1* were named *RcWRI1-A* and *RcWRI1-B*, respectively.

In order to verify the two alternate splice forms of RcWRI1, we aligned the RcWRI1 genomic sequence and two RcWRI1 cDNA sequences (Figure S1). The genomic sequences that match the cDNA are exons, and the alignment gaps are introns. Putative intron boundaries conform to the GT-AG rule [30]. Exon 3 consists of 9 bp in *RcWRI1* gene, which is spliced out in *RcWRI1-B* where exon 2 directly links to exon 4. Thus, *RcWRI1-B* is 9-bp shorter than *RcWRI1-A* (Figure S2). Therefore, *RcWRI1-A* and *RcWRI1-B* are the alternative splice forms from the same *RcWRI1* gene.

**Figure 2:**
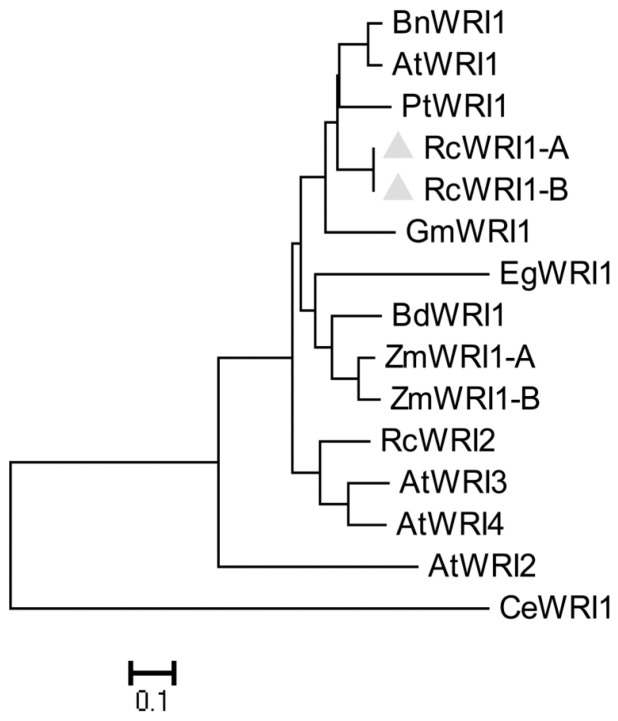
Phylogenic tree of RcWRI1s and WRI1 homologous from other plant species. Phylogenetic tree was generated using MEGA6 by the neighbor-joining method. Percentage values on each branch represent the corresponding bootstrap probability. Protein sequences used for phylogenic analysis included BnWRI1 (ADO16346), ZmWRI1-A (ACG32367.1), ZmWRI1-B(AIB05036.1), CeWRI1 (SRX1079431), PtWRI1 (XP_002311921.2), GmWRI1 (XP_006596986.1), BdWRI1 (Brai4g43877), AtWRI1 (AAP80382), AtWRI2 (ABG25074), AtWRI3 (NP_001320852.1), AtWRI4 (ABK32182.1), RcWRI2 (BAM75179.1) and EgWRI1 (AHX71676.1). The two RcWRI1s protein are highlighted by the grey arrowheads.

Further alignment of RcWRI1-A, RcWRI1-B and AtWRI1 indicated that these three proteins share 90% sequence similarity in the two highly conserved AP2 domains at their N-termini (Figure 1). However, the C-terminal regions are highly diverged, with only 31% identity. In addition, the sequence “VYL” present in the first AP2 domain of WRI1 protein, which is essential for its function, is conserved in AtWRI1 and RcWRI1-A, whereas this important motif was absent in RcWRI1-B (Figure 1). To clarify the phylogenetic relationship between the two RcWRI1 isoforms and AtWRI1, we included the related *Arabidopsis* proteins AtWRI2, AtWRI3, and AtWRI4, as well as other WRI homologous proteins from different species. Phylogenetic analysis by MEGA6 showed that RcWRI1-A and RcWRI1-B was the closest to AtWRI1 (Figure 2), because they fell into the same clade as AtWRI1 and BnWRI1. AtWRI1 is the only one responsible for TAG accumulation in seeds among members of AtWRI1 family [16].

### 2.2. Expression Profiles of RcWRI1-A and RcWRI1-B in Various Castor Organs

**Figure 3:**
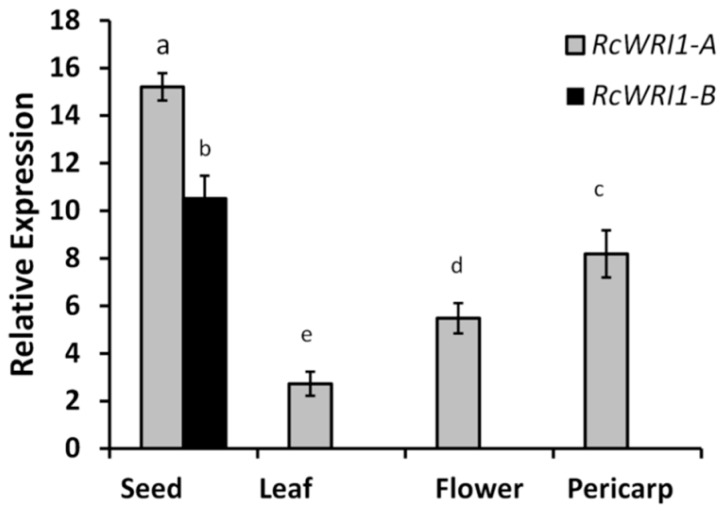
Expression of *RcWRI1-A* and *RcWRI1-B* in major castor bean organs. Expression profiles were determined by qRT-PCR using total RNA from leaves, flowers, pericarps, and developing seeds. *RcActin* was used as an internal control. Each value is the mean ± SD of six biological replicates. Letters a, b, c, d, e indicate significant differences at the level of *p* < 0.05 according to the Tukey’s test.

We assessed the expression of *RcWRI1-A* and *RcWRI1-B* in different organs of castor bean plants by quantitative RT-PCR (qRT-PCR). The transcript-specific primers (Figure S3A) were designed to distinguish *RcWRI1-A* from *RcWRI1-B*. *RcWRI1-A* was expressed in all organs tested (Figure 3), with the highest expression in developing seeds (30 DAF, days after florescence) and the lowest in the leaf. However, *RcWRI1-B* was only expressed in developing seeds. These results indicate that both *RcWRI1* isoforms are actively transcribed during seed development. Similar to *AtWRI1* in *Arabidopsis* siliques [12], the predominant expression of *RcWRI1-A* and *RcWRI1-B* in castor seed suggests that these two castor *RcWRI1* isoforms function importantly in seed development. *RcWRI1-B* is unlikely to play a biological role in castor leaf, flower, and pericarp because of no expression was detected in these organs.

### 2.3. Overexpression of Individual RcWRI1 Splice Forms Restored Seed Total Lipid Content in the Arabidopsis wri1-1 Mutant

**Figure 4:**
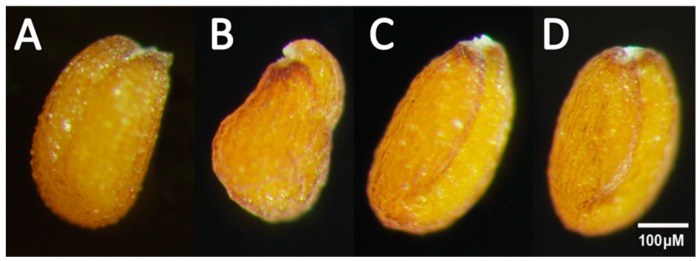
*RcWRI1-A* and *RcWRI1-B* complemented the *Arabidopsis* wrinkled seed phenotype. The seed phenotype was examined by a stereo microscope. (**A**) Wild type; (**B**) *wri1-1* mutant; (**C**) *wri1-1* expressing *RcWRI1-A*; (**D**) *wri1-1* expressing *RcWRI1-B*.

**Figure 5:**
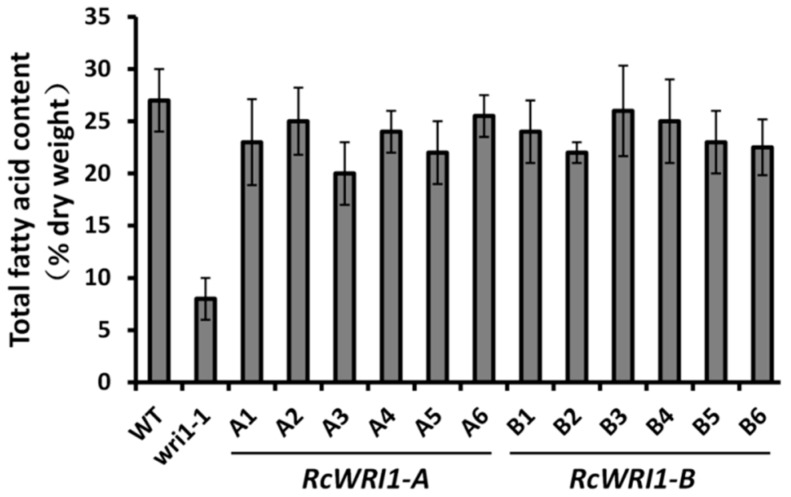
Total fatty acid content in seeds of *Arabidopsis* wildtype, *wri1-1* mutant, and the mutants expressing either *RcWRI1-A* or *RcWRI1-B*. Total fatty acids were extracted from the seed and transmethylated, followed by gas chromatography analysis. Fatty acid content was expressed as the percentage of seed dry weight. Each value is the mean ± SD of six biological replicates.

In order to investigate the function of the *RcWRI1s*, we separately overexpressed *RcWRI1-A* and *RcWRI1-B* under a seed-specific Gly promoter in the *Arabidopsis wri1-1* loss-of-function mutant. A microscopic observation of mature dry seeds showed that transgenic *wri1-1* plants expressing *RcWRI1-A* or *RcWRI1-B* displayed a non-wrinkled phenotype (Figure 4). We further measured the fatty acid content in seeds of transgenic lines. Overexpression of *RcWRI1-A* or *RcWRI1-B* restored total fatty acid content in seeds to the wild-type level (Figure 5).

### 2.4. Transient Expression of RcWRI1s Enhances the Expression of the Downstream Genes in the Fatty Acid Biosynthesis Pathway

**Figure 6:**
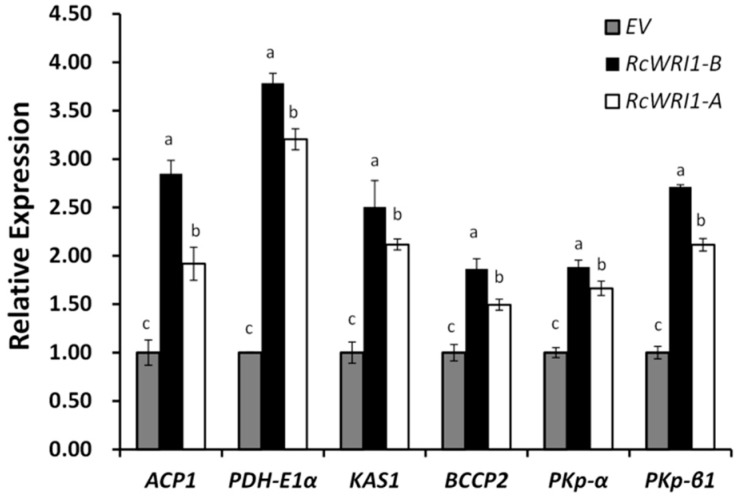
Expression profiles of WRI1 target genes in tobacco leaves expressing either *RcWRI1-A* or *RcWRI1-B*. Total RNA was isolated from the leaves expressing each of *RcWRI1s*, and used for qRT-PCR analysis. The primers were designed based on the coding sequences of *PKp-β1*, *PKp-α*, *ACP1*, *PDH-E1α*, *BCCP2*, and *KAS1* of *N. benthamiana*. EV indicates empty-vector control. *NbActin* was used as an internal control. Each value is the mean ± SD of six biological replicates. Letters a, b, c indicate a significant difference at the level of *p* < 0.05 according to the Tukey’s test.

To investigate the function of RcWRI1-A and RcWRI1-B in gene regulation, we further examined the expressions of the putative target genes in *N. benthamiana* leaves where *RcWRI1-A* or *RcWRI1-B* were transiently expressed (Figure S3B). These putative target genes include plastid pyruvate kinase beta subunit 1 (*PKp-β1*), pyruvate kinase alpha subunit (*PKp-α*), acyl carrier protein 1 (*ACP1*), pyruvate dehydrogenase E1 component alpha subunit (*PDH-E1α*), biotin carboxyl carrier protein isoform 2 (*BCCP2*), and 3-ketoacyl-acyl carrier protein synthase 1 (*KAS1*) [14,15]. qRT-PCR analysis revealed that expression of these target genes was significantly increased in leaves either expressing *RcWRI1-A* or *RcWRI1-B* relative to the empty-vector (EV) controls (Figure 6). Notably, expression of *PDH-E1α* was three times higher than in the control. Moreover, *RcWRI1-B* appeared to show a stronger activity in promoting the target gene expression than *RcWRI1-A*.

### 2.5. Ectopic Expression of RcWRI1s Increases Total Lipid Content in N. benthamiana Leaves

**Figure 7:**
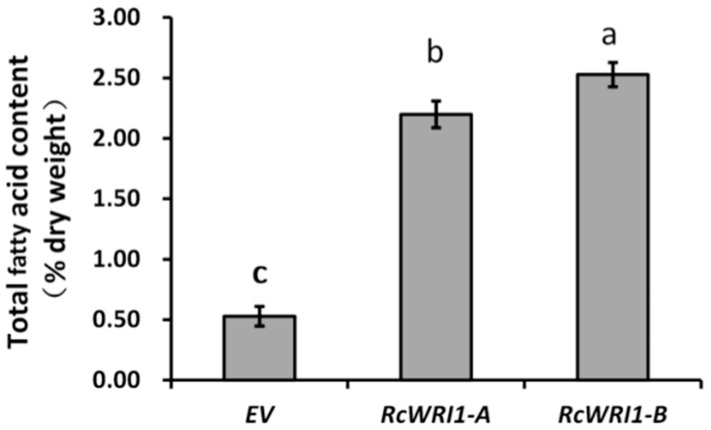
Total fatty acid content in *N. benthamiana* leaves expressing each of *RcWRI1s*. Fatty acids were extracted from the leaf samples and then transmethylated, followed by gas chromatography analysis. Fatty acid content was expressed as the percentage of leaf dry weight. EV indicates empty-vector control. Each value is the mean ± SE of twelve biological replicates. Letters a, b, c indicate a significant difference at the level *p* < 0.05 according to the Tukey’s test.

To characterize the function of *RcWRI1-A* and *RcWRI1-B* in fatty acid biosynthesis and oil accumulation, we tested whether the expression of each castor *RcWRI1* isoform could lead to the increase of the total lipid content in transformed *N. benthamiana* leaves. For this purpose, we expressed the genes under the control of the CaMV35S promoter through *Agrobacterium*-mediated transformation. The infiltrated *N. benthamiana* leaves were subjected to the analysis of total lipid content. As shown in Figure 7, total lipid content in the leaves expressing each of *RcWRI1s* was remarkably higher than that in the control leaves infiltrated with the empty vector, with leaves expressing *RcWRI1-B* showing a higher level of oil concentration (2.5% of leaf dry weight). These results indicate that both *RcWRI1* isoforms function actively in induction of fatty acid/oil accumulation in heterogenous leaves when transiently expressed. Again, *RcWRI1-B* is more active than *RcWRI1-A* in this regard.

### 2.6. Ectopic Expression of RcWRI1s Changes Fatty Acid Profiles in N. benthamiana Leaves

**Figure 8:**
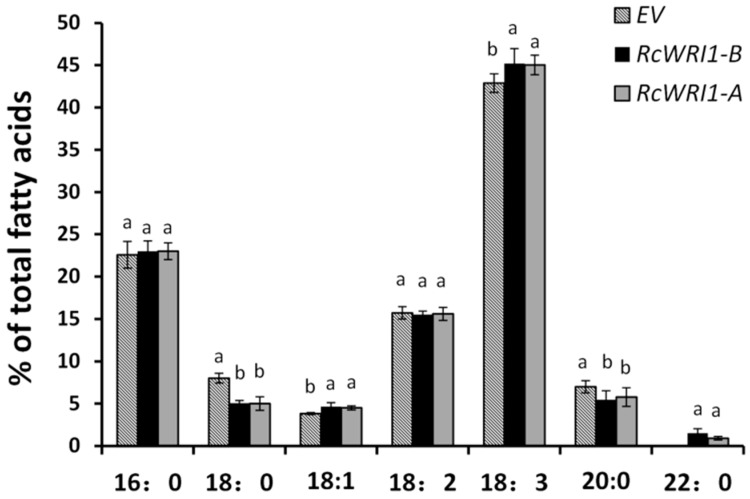
Fatty acid profiles in *N. benthamiana* leaves expressing each of *RcWRI1s*. Fatty acids were extracted from the leaf samples and then transmethylated, followed by gas chromatography analysis. Fatty acid content was expressed as the percentage of leaf dry weight. EV indicates empty-vector control. Each value is the mean ± SE of twelve biological replicates. Letters a, b indicate significant differences at the level *p* < 0.05 according to the Tukey’s test.

To examine the effects of *RcWRI1* expression on fatty acid composition, we further analyzed the fatty acid profiles in *N. benthamiana* leaves transformed with *RcWRI1-A* or *RcWRI1-B*. As shown in Figure 8, expression of either *RcWRI1-A* or *RcWRI1-B* induced a subtle change in the fatty acid composition in the leaves, such as the increases in 18:1, 18:3 and 22:0 fatty acids when compared with the controls. However, 18:0 and 20:0 contents were reduced in the *RcWRI1*-expressed leaves. No obvious change was detected for the 16:0 and 18:2 fatty acid contents between the *RcWRI1*-expressing leaves and the controls. No significant difference in this regard was detected between *RcWRI1-A* and *RcWRI1-B*. Unexpectedly, a new long chain fatty acid 22:0 was synthesized and accumulated in the leaves expressing each of *RcWRI1s*, but not in the vector controls. The possible reason may be that *RcWRI1* overexpression increases the activity of fatty acid elongases (*FAE*) [18].

## 3. Discussion

### 3.1. Castor RcWRI1 Expresses Two Alternative Splice Forms with Tissue-Specific Patterns

Multiple isoforms of *WRI1* were recently identified despite the majority of plants were detected to have a single *WRI1* gene. For example, maize genome contained two isoforms, *ZmWRI1a* and *ZmWRI1b* [20]. Three functional *CsWRI1* isoforms (*CsWRI1A*, *B*, and *C*) were discovered in camelina genome [21]. In addition to *AtWRI1*, *AtWRI2*, *3*, *and* *4* were identified to have the similar function as *AtWRI1* despite of their different expression patterns [16,23]. The isoforms from a plant species exhibited a very high sequence homology are consistence with gene duplication in polyploid genomes such as camelina experienced the whole genome triplication event. These isoforms also provide the targets for evolution selection to increase their functional divergence, which is suggested by their different expression patterns detected in various tissues/organs.

In eukaryotic cells, many genes can increase their functional diversity by alternative splicing [31,32]. For *AtWRI1*, three alternative splice forms were predicted, but only the *WRI1* splice form 3 (At3g54320.3) transcript accumulated in multiple *Arabidopsis* tissues, including roots, flowers, developing seeds, and young seedlings [23]. RNAseq (RNA Sequencing) and EST (expressed sequence tag) data available for *Brassica napus* showed that the isoform corresponding to the *WRI1* splice form 3 was only expressed in developing seeds [23]. In this study, two splice forms of *RcWRI1* were cloned from castor bean, and their sequences were identical except for *RcWRI1-A* having a 9-bp fragment (TTTATTTGG) which is absent in *RcWRI1-B* (Figure 1). Unlike the three *AtWRI1* splice forms, both splice forms of *RcWRI1* were highly expressed in castor developing seeds (Figure 3). Moreover, *RcWRI1-A*, but not *RcWRI1-B*, is also expressed in the castor flower, leaf, and pericarp. The different expression patterns of these two *RcWRI1* splice forms indicate that *RcWRI1-A* and *RcWRI1-B* may function in a tissue-specific manner although further studies are needed to characterize their functional divergence.

### 3.2. The Motif “VYL” Encoded by a 9-bp Exon Is an Important Component of WRI1 Proteins, But Not Necessary for Function of All WRI1s

Previous data showed that WRI1 proteins are conserved in regulating plant oil biosynthesis [16,21,25]. Moreover, The WRI1 orthologs and homologs show a high level of amino acid identity in the region spanning the two AP2 domains but with a high degree of divergence in the N-termini and C-termini [23,26]. The C-terminal WRI1 activation domain is crucial for transcriptional activation. However, the C-terminal region is very different in AtWRI1 and RcWRI1. In the first AP2 domain responsible for DNA-binding, a short motif “VYL” encoded by the 9-bp exon was conserved in AtWRI1 splice form 1 and 3, as well as other WRI1 orthologs from diverse plant species [33,34]. Of the 34 predicted AtWRI1-orthologous proteins at Phytozome, only two contained “IYL” instead of “VYL” [23]. Single amino acid mutation for each of “VYL” resulted in impairment of AtWRI1 function [23], indicating that “VYL” is an essential motif for function of AtWRI1. However, the “VYL” motif is missing in a number of WRI1 orthologs [23] although their functions have not been characterized yet. In the present study, the “VYL” is present in RcWRI1-A but absent in RcWRI1-B (Figure 1). Transient expressions of either *RcWRI1-A* or *RcWRI1-B* in *N. benthamiana* leaves led to a significant increase of total FA content in the leaf tissue (Figure 7), suggesting that the lack of “VYL” do not affect the function of RcWRI1-B in promoting lipid biosynthesis and oil accumulation. Therefore, the “VYL” motif is not necessary for all WRI1 proteins.

### 3.3. Castor RcWRI1-A and RcWRI1-B Function to Increase Oil Biosynthesis in Heterogenous Tobacco Leaves by up Regulating the Target Genes Involved in Glycolysis and FA Synthesis

To meet the increasing demand for plant oils as foods and non-food uses, engineering high-biomass leaves for oil production is an alternative and promising strategy to increase the production of vegetable oils [1,35,36]. Transcriptional factor WRI1 is one of targets used in such approaches. For example, overexpression of *AtWRI1* alone increased TAG content to 2.8-fold in *Arabidopsis* seedlings without obvious negative effects on plant development [36]. Similarly, ectopic expression of *WRI1s* from diverse species including potato, poplar, oat, nutsedge and camelina enhanced TAG levels by approximately 2–4-fold in *N. benthamiana* fresh leaves relative to the control leaves [21,26]. All these WRI1s function to induce oil accumulation in leaves via upregulating the genes encoding the enzymes required for FA biosynthesis, such as *PKp-β1*, *ACP1*, *PDH-E1α*, *BCCP2*, *ACCase*, *SUS2*, and *KAS1* [21,26,36]. 

It was evident that ectopic expression of *BdWRI1* isolated from a cereal plant *Brachypodium distachyon* induced an enhancement of TAG content in *B. distachyon* leaves, but simultaneously caused cell death in the leaf blades because of increased free FAs resulted from TAG turnover [25]. This negative phenotype by *BdWRI1* overexpression in vegetative tissues was not observed for other *WRI1s*, indicating that the specific phenotypes caused by ectopic expression of *WRI1* are dependent on species context although most of *WRI1* overexpression experiments did not show negative effects.

Here, we transiently expressed castor *RcWRI1-A* and *RcWRI1-B* in *N. benthamiana* leaves. As expected, expressing either of these gene isoforms greatly upregulated the expressions of several WRI1 target genes, and subsequently resulted in a drastic increase of total FA content in the transformed leaf tissue. Furthermore, RcWRI1-B lacking the “VYL” motif displayed a much stronger activity than RcWRI1-A. Taken together, these data confirm that both RcWRI1-A and RcWRI1-B have a conserved function in regulating FA and TAG biosynthesis. Ectopic expression of *RcWRI1s* or other *WRI1s* can be a potential tool for increasing the energy density in vegetative organs of plants.

## 4. Materials and Method

### 4.1. Plant Materials

Castor bean (*Ricinus communis* L.) plants were grown in a greenhouse with 12/12 hr light/dark photoperiod at temperatures between 18 and 28 °C. *Nicotiana benthamiana* seedlings in a sterilized soil mixture (peat moss-enriched soil:vermiculite:perlite in 4:2:1 ratio) were grown in a growth chamber under fluorescent light (200 μmol∙m^−2^∙s^−1^) and with the following conditions: 16/8 h light/dark, 21/25 °C, and 50–60% humidity. Wild-type *Arabidopsis thaliana* and the *wri1-1* mutant plants were grown in a growth chamber at 21/25 °C (day/night) with a 16 h light/8 h dark.

### 4.2. Castor Bean RcWRI1 Cloning

The *RcWRI1* gene of castor bean (LOC8283400) was identified by searching the castor bean genomic database (Available online: http://castorbean.jcvi.org/index.php) using the BLAST algorithm and *Arabidopsis* AtWRI1 (At3g54320) sequence as query sequence. ORF of this gene was used to design a pair of primers to amplify *RcWRI1* coding sequence. The primers used were *RcWRI1*-F1: 5′-ATGAAGAGGTCTCCTACTTC-3′ and *RcWRI1*-R1: 5′-TCAAACAGAATAGTTA CAAC-3′. Developing seeds (30 day after flowing, DAF, of castor bean were sampled to isolate total RNAs, which were then used for first-strand cDNA synthesis by PrimeScript™ RT reagent Kit (Takara, Dalian, China). The cDNAs were subsequently used for amplification of RcWRI1 ORF by RT-PCR using EasyPfu DNA Polymerase. Two amplicons different in length were cloned into pGEM T-easy vector (Promega, Madison, WI, USA) and their nucleotide sequences were determined by sequencing. The long form (1341 bp) was designated as *RcWRI1-A* while the short form (1332 bp) was named *RcWRI1-B*.

### 4.3. Sequence Alignment and Phylogenetic Analysis

Multiple alignments of amino acid sequences of RcWRI1-A, RcWRI1-B and other plant WRI1s were performed with the ClustalW program (Available online: http://www.ebi.ac.uk/Tools/msa/clustalo/). Phylogenetic analysis was conducted by MEGA6 program with the Neighbor-joining algorithm method and a bootstrap value of 1000 replicates.

### 4.4. Vector Construction

**Figure 9:**
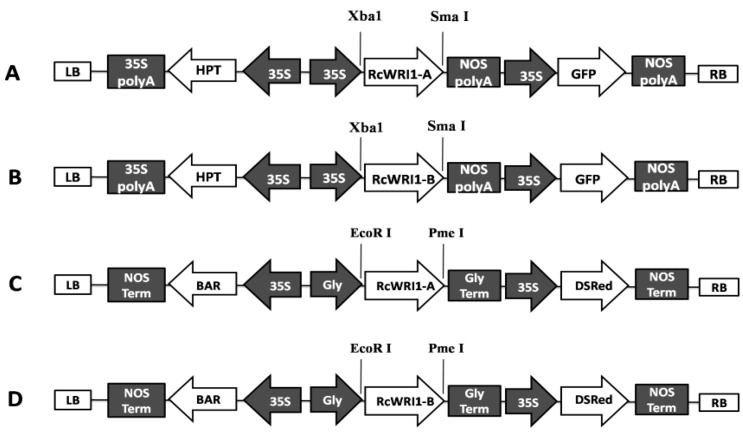
Schematic representation of *RcWRI1-A*, and *RcWRI1-B* expression constructs used for transient expression in *Nicotiana benthamiana* leaves and overexpression in the *Arabidopsis wri1-1* mutant. (**A**) The *RcWRI1-A* expression cassette in pCAMBIA1303. (**B**) The *RcWRI1-B* expression cassette in pCAMBIA1303. (**C**) The *RcWRI1-A* expression cassette in pJC-Gly-DsRED. (**D**) The *RcWRI1-B* expression cassette in pJC-Gly-DsRED. 35S: CaMV 35S promoter. HPT: Hygromycin resistance gene. 35S polyA: 35S poly (A) signal sequence. Nos polyA: Nopaline synthase poly (A) signal sequence. Gly: Gly-promoter. BAR: Herbicide resistance bar gene. Gly term: Gly terminator. NOS term: Nopaline synthase terminator. GFP: Green fluorescent protein. RB: Right border. LR: Left border. DSRed: Discosoma red fluorescent protein.

The ORFs of *RcWRI1-A* and *RcWRI1-B* were separately amplified from their corresponding clone vectors using gene-specific primers with Xbal (5′-end) and SmaI (3′-end) sites (*RcWRI1*-F2: 5′-GCTCTAGAATGAAGAGGTCTCCTACTTCC-3′ and *RcWRI1*-R2: 5′-TCCCCCGGGTCAAACA GAATAGTTACAAC-3′). *RcWRI1-A* and *RcWRI1-B* ORFs were then respectively inserted behind the CaMV 35S promoter in the pCAMBIA1303 vector (Figure 9). This vector contains a reporter gene GFP driven by the CaMV35S promoter, which facilitates the identification of transgenic events in transformation of *Nicotiana benthamiana*. For transformation of *Arabidopsis*, the ORFs of *RcWRI1* were amplified from clone vectors using primers containing EcoRI and PmeI sites (*RcWRI1*-F3: 5′-CGGAATTCATGAAGAGGTCTCCTACTTCC-3′ and *RcWRI1*-R3: 5′-GGGTTTAAACTCAAA CAGAATAGTTACAAC-3′). *RcWRI1s* was then transferred into the pJC-Gly-DsRED binary vector under the control of the seed-specific Gly-promoter and with the DsRed selection marker. These vectors containing *RcWRI1-A* or *RcWRI1-B* were then transformed into *Agrobacterium tumefaciens* GV3101 by the freeze-thaw method, which were used for *Arabidopsis* plant transformation.

### 4.5. Overexpressing RcWRI1s in the Arabidopsis wri1-1 Mutant

The expression vectors pJC-Gly-DSRED-*RcWRI1s* were introduced into the *wri1-1* mutant through *Agrobacterium tumefaciens*-mediated transformation by floral dipping [37]. Overnight culture of *Agrobacterium* was harvested by centrifugation, and then resuspended in infiltration medium with OD600 at 0.80 prior to use. The infiltration medium contained 5.0% sucrose and 0.05% Silwet L-77 (Solarbio, Beijing, China). *Arabidopsis* plants were inverted into the beaker of infiltration buffer and held vacuum for 10s in a vacuum chamber. After that, the plants were removed to a plastic tray and covered with a clear plastic for overnight in dark. Finally, the plants were returned to the growth chambers. The seeds were selected on MS (Murashige and Skoog) medium containing PPT (phosphinothricin) (10 mg/L) (Sigma-Aldrich, Darmstadt, Germany) and transferred to soil for further characterization. Homozygous plants were subsequently used in all experiments.

### 4.6. Transient Expression in Tobacco Leaves

**Figure 10:**
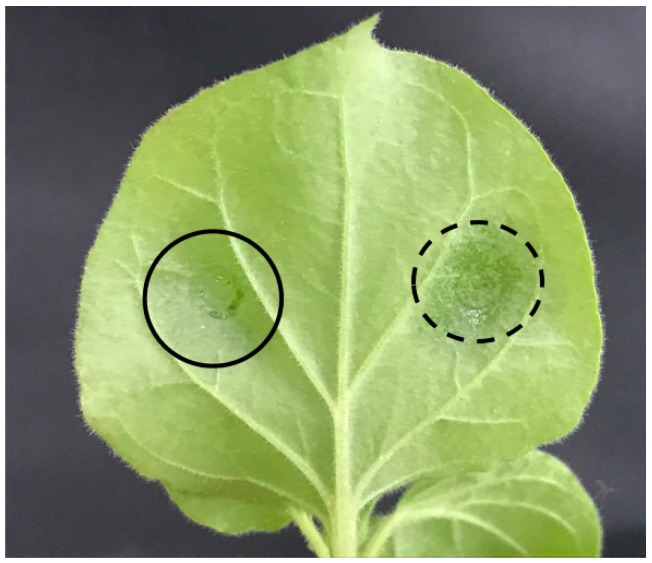
Tobacco leaf image showing the regions infiltrated by the *Agrobacterium*. The left-half part of the leaf was infiltrated with *Agrobacterium* containing the *RcWRI1* expression vector, while the right-half part of the leaf was infected by *Agrobacterium* containing an empty vector. The solid black circle shows the region infiltrated by the target gene, and dotted black circle marks the region infected by the empty-vector control.

*Agrobacterium tumefaciens* GV3101 harboring the *RcWRI1* gene expression vector was cultured overnight, and then resuspended in the buffer solution (100 μmol/L acetosyringone (Sigma-Aldrich, Darmstadt, Germany), 10 mmol/L MgCl2, 10 mmol/L MES (MES monohydrate) (PhytoTechnology, Shawnee Mission, KS, USA) to prepare the bacterium mixture with OD600 at 0.125 prior to infiltration. Tobacco (*N. benthamiana*) leaves of the 6-week-old plants were selected for agroinfiltration (Figure 10). One leaf per plant representing the same development stage was selected for infection. Six biological replicates were conducted for each expression vector. Three hundred microliters of the bacterium culture mixtures were infiltrated into the leaf, and plants were then incubated for six days in growth chambers with the same growth conditions as described above. The infiltrated leaf area showing *GFP* expression under UV-light were sampled, immediately frozen in liquid nitrogen, and then stored at −80 °C. The samples were used for RNA extraction (~200 mg FW (fresh weight)) and for lipid analyses (~800 mg FW). The empty-vector infected leaf areas were sampled as controls. 

### 4.7. Real-Time PCR to Examine Expression of RcWRI1-A and RcWRI1-B

Total RNA from castor flowers, leaves, pericarps, developing seeds (30 DAF) and tobacco leaves was extracted using Trizol Reagent (Shenggong BBI Life, Shanghai, China). One microgram of total RNA was used to synthesize the first-strand cDNA by PrimeScript RT reagent Kit with gDNA Eraser (Takara, Dalian, China). qRT-PCR was performed using the SYBR Premix Ex Taq II (Takara) on CFX 96 Real-Time PCR Detection System (BIO-RAD, Hercules, CA, USA), using a program with initial 95 °C for 30 s followed by 40 cycles of 95 °C for 5 s, and 60 °C for 30 s. Primers for qPCR are shown in Table 1. The 2^−ΔΔ*C*t^ calculation was used to determine the relative expression of downstream genes of WRI1, and two *RcWRI1* expression levels in various castor organs calculated by 2^−Δ*C*t^ [38].

### 4.8. Lipid Analysis

To measure total fatty acid (FA) profile and content, total lipids extracted from leaf samples collected from *N. benthamiana* plants and *Arabidopsis* seeds. Tri 17:0-TAG in chloroform was added into sample as the internal control. Freeze-dried leaf and seed samples were ground into powder and 20 mg powder was used for total lipid extraction by following method described by Li et al. [39]. Briefly, the samples were homogenized in 1 mL chloroform and methanol (*v*:*v* = 2:1) containing 0.001% BHT, followed by adding 0.5 mL 0.9% (*w*/*v*) KCl solution and 1 mL chloroform, and then spinned for a few minutes for phase separation. The lower clear liquid phase was transferred into a clean glass tube, and the sample was dried by N_2_ flow. After that, 0.5 mL sodium methoxide was added into the tube for FA esterification, with shacking for 30 min. Finally, 0.5 mL isooctane was added into the esterified samples with well mixture. The upper layer containing the FA methyl esters (FAME) was transferred into GC auto-sampler vials. The samples were measured by gas chromatography (Agilent 7890B, 30 m × 0.25 mm × 0.25 μm FFAP(free fatty acid phase) column, flame ionization detector, (Agilent Technologies, Santa Clara, CA, USA)). The peak area for each FA on the retention time was characterized and measured by comparison with the known standard FA profiles, and the concentration of each FA was calculated by comparing its peak area with that of the internal standard.

## 5. Conclusions

The *RcWRI1* gene in castor expressed two alternative spliced forms, *RcWRI1-A* and *RcWRI1-B*. These two splice forms exhibited tissue-specific expression patterns in castor. Expression of either *RcWRI1-A* or *RcWRI1-B* could rescue the low oil content in the *Arabidopsis wri1-1* seeds. Transient expression of either *RcWRI1-A* or *RcWRI1-B* in *N. benthamiana* leaves significantly upregulated the expressions of the target genes such as *PKp-β1*, *ACP1*, and *KAS1*, and greatly increased total FA levels in the leaves, indicating that both gene isoforms function in regulation of FA biosynthesis and TAG accumulation. The conserved “VYL” motif is not necessary for function of all WRI1 proteins including RcWRI1-A and RcWRI1-B although it is essential for *Arabidopsis* AtWRI1 function. The present findings demonstrate that *RcWRI1-A* and *RcWRI1-B* can be employed as master transcriptional factors to engineer high-biomass plants for increasing commercial production of vegetable oils.

## Figures and Tables

**Table 1:** Primer sequences used for qRT-PCR in this study.

Primer Name	Forward Primer Sequence (5′-3′)	Reverse Primer Sequence (5′-3′)
***NbActin***	CAGTGGCCGTACAACAGGTA	AACCGAAGAATTGCATGAGG
***NbPKp-β1***	CTGTGTCGCTACGGACTGAA	GTGTTGGACATCATCGTTGC
***NbPKp-α***	TAAAAGCTCGGGGCATGGTC	GGTTTCCCAGCTAAGAACTTGT
***NbPDH-E1α***	TGGTAACGATGCTCTTGCTG	CTCCACCATTCGATTTCGTT
***NbACP1***	GCAAATGGATCGAGGCTAAC	CTTGGAAGGATCGACTTTGG
***NbKAS1***	CACCCATCAATTCACCAATG	CCCATCCCAGTTATGACCAC
***NbBCCP2***	GCTGATTCGTCTGGAACCAT	GCCTTCCACCTATGTTGCAT
***RcActin***	GTGCTTGATTCTGGTGATGGC	TTGGCAGTCTCAAGTTCTTGCTC
***RcWRI1-A***	GAAGGGAAGACAAGTTTATTTGG	ATTCAAGGTTGTCTCTGGTCC
***RcWRI1-B***	AAGGGAAGACAAGGGGCCTATG	GCTTTGGCGTCGAAGAGATG

## Supplementary Materials

Supplementary materials can be found at www.mdpi.com/1422-0067/19/1/146/s1.

## Abbreviations

TAGs	triacylglycerols
ORF	open reading frame
PKp-β1	plastid pyruvate kinase beta subunit 1
PDH-E1α	dehydrogenase E1 component alpha subunit
BCCP2	biotin carboxyl carrier protein isoform 2
ACCase	acetyl-CoA carboxylase
SUS2	sucrose synthase 2
KAS1	3-ketoacyl-acyl carrier protein synthase 1
Pyr	pyruvate
CoA	acetyl-coenzyme A
ER	endoplasmic reticulum
G3P	glycerol-3-phosphate
DAF	days after florescence
FFAP	free fatty acid phase
FAME	FA methyl esters
MS	Murashige and Skoog
PPT	phosphinothricin
MES	MES monohydrate
FW	fresh weigh
EST	expressed sequence tag
