# Supplementary material for: Splice Variants of the Castor WRI1 Gene Upregulate Fatty Acid and Oil Biosynthesis When Expressed in Tobacco Leaves
Source: Int J Mol Sci. 2018 Jan 5;19(1):146. doi: 10.3390/ijms19010146 (PMC5796095; doi:10.3390/ijms19010146)
Supplement: Supplementary file 1 [file ijms-19-00146-s001.zip › Figure S1 .pdf]

>NW_002994462.1

1 GCATAAACAA TGAATATTTA ATATATATTT AATAAACATA TATTATTGAA TATTTAGTAT

61 AAATTTAATA AAATATAATT TTTTTTTTAA AAAAAATTCA TATAAAGATC AGACGCATAT

121 CACATCACGT GCACATCAAT ATTTGACCCA CGTATATACA TGGATTAGAG CATATAGCTT

181 CATTCAACAG GTTCCAACTA AATCTCCCGC CATGGTATGC TACCGCTGCC TAAACGACAA

241 CTCGTTTATG TCGCAATTTG TAAAGCTGTC TGATGAGCAA CCAAAATAAA ATGCAAAAGA

301 AAAAAAAAGG AAAAAAGAAA ACAAAGAGGA GGTTTAAGTC ATTATGTGTA TAGATTGTAG

361 AACTCCTTAA AAATAACTCA GAAATGTCCT TACACGTCCC TTAGTTTTCA AGTGAAACAC

421 AGACACAGAG CCAGAAAAAC ACAAAGTAAC TGACATATCA GTGATATAAA AAATAAAACT

481 TAAAAGAAAG AAGGGATTAA GAAATTAAAA AGTCATTTAT TTAAAATAAT AGTGAGATTC

541 TGTCAAACTA CTTACAAGGC CAGCAAGCAT AGGGCTAAAA ACACCCGATT GGTAGCATGG

601 CTGCAGCTTA AGCCAAGTGT TGGGCATTAA TTTAACTCAT GGTTAACTAT TAACCCTAAC

661 TCTTCTTAAC TGCTGTTTGC CCCCATGGTT AAACTACAAC AACATGTTTT GCACTCATTT

721 TGGGCAGGGT TTTTCCTTCT TCTTTTTTTT TGGGTAAATT AAAAATAATA ATAAATACCT

781 TTCGCTTCTG CTTCTCCCCA TTGTCTCTCC ACTTCTTGTT TTGGTAAACC ACTTCTGGTT

841 ACCAAAAAAA ACCCACCTCT CTTACATTCT TCAAAGTCGC AAAATCTTTA CCAAAAGAGA

901 GAGAGAGAGA GACCTAACTT AAAAAAATCA TACTCTTCTT GAGTACTGCA TTTAAGTTTC

961 TTTTGTTTTG TTTCTCTCAT TAAAACAAGA AGAAGAACTT AAGAAGAAGA AGTAAATGAA

1021 GAGGTCTCCT ACTTCCCCGT GCTCTTCTTC TTCATCATCT TCATATTCAT CTTCTTCAGC

1081 TTCATCTTCT TGTGTCGGTC CTGATGATAC TCCTGTCGCT CCTGGTAGTC ATCATCATCA

1141 TGATCACCAC CAATTACGAT CGCAGAAATC ATCCAAACGC ATTAGAAAAG TTAAAAAAAA

1201 GCAGCAGAAC CATAATATTG ATCAGAATAA TACTAACACC ACTATTACTG CTCCTACCTC

1261 TGCCAGAAGA AGCTCCATTT ACAGAGGAGT CACCCG**GT**AC TTGCTACTAT TATATATTTA

1321 TATATATATA TATATATATA TATTCTAATA CCAATTGGCT AATTGCTGGG ACTAATTGAC

1381 TAATATTTCC TATAATGTAT ATTTTGTTTT CTGGCTTTTC ATTATTATTA TTATAAAAAT

1441 TTTGTGTTGA TGAAAAC**AG**G CATAGATGGA CGGGAAGGTT TGAGGCACAC CTTTGGGATA

1501 AGAGTTCATG GAATAACATT CAAAACAAGA AGGGAAGACA AG**GT**GATTTT CCCTTAATTA

1561 ATTAATAATT ATTTTATAAT GATTATTATT TTGTTTTTAC AAAAATCAAG GAGATCTGTA

1621 ATATTAATTA TTTAATAAAT TATTTTACTT TTTTAGAGAC TTTTCTTTCA TATTTGTTTT

1681 TCTTAATGTT TTCTAATAAC TTATTGTTAT GTTCTGACTA CGTTTTATTC CTGATTATCC

1741 CTGTCACTGT AACGTTCATG TACACTTTTG TCGCTGCTT**A G**TTTATTTGG**GT**AAGCACTT

1801 ATGACCTCAA ACCAATATTT TAATCACTTT CATTTTATTT TTTAGTAATA AATGATTATT

1861 CTTTTTTATT TTCTAATTGA AAAAATAAAA TTGC**AG**GGGC CTATGATAAT GAAGAAGCAG

1921 CTGCTCATAC TTATGACCTT GCTGCCCTTA AGTACTGGGG ACCAGAGACA ACCTTGAATT

1981 TCCCC**GT**ATG ATAAACTTTT TTTTTATATT AATTGTCAAT ATTTAAAAAG CAAAAACGTA

2041 CTCAATTGGG GTGAATTGGG TGTTGT**AG**AT AGAAACATAT CCTAAAGAGC TTGAAGAGAT

2101 GCAAAAGATG AGCAAGGAAG AGTACTTGGC ATCTCTTCGA CGCCAAAGCA GTGGGTTTTC

2161 CAGAGGAGTT TCTAAGTACC GTGGGGTTGC TAG**GT**AATTT CTCTTTTTTC TTTTTTGTTT

2221 TTCGAGAAGG ACAAAGAAAT AAATAAACTC GAACATAAGA TTTTATTCAA TTTTCTTTGA

2281 ACATTCGAGC TTGAAATTTT CTTACTTAAT TTCAAATATA CACTGCTGTC AGGCTAAGTC

2341 CGTTGATGCA TTAATAAAAT AATATTAAAT TTGCCTTTAA TTAAATTTGT TTTATTGGTT

2401 TGAAATTCTG TAAC**AG**GCAT CACCACAATG GTCGATGGGA GGCACGAATC GGACGAGTTT

2461 TTGGCAACAA GTATCTCTAC CTTGGCACTT ATA**GT**ACGCT AATCTCTCCT TTCTACAAAT

2521 AATCTTCTTT TATTAGAACG TCAAAATGCT ACTAATCTAA TTATGGAATA AAAGAAGTGG

2581 GCTGTGCAAC CTGAATATAT AGGAATGAGC AATGCCTAAT TAGGAAATAA TCAATGATAT
2641 CATCATCATA GTAAACTTGG CCCCCGCCTC AATTAATTAG ACACTAGAAT GCTTACCTAC
2701 CTTAATTCAT CTCATTTAAC ACATTTATTT GACCCGTAAT TTTTAATTAT ACCAAAAATA
2761 TATTAAATAT AGATTCTTAA TAAATAGATG AATGATATAT TTACATTATT ATTCTATATT
2821 ACTATATTTA ATATTAAATA ATTAATATAA TTAATTATTT AAAATCAAGA AATATGTGTA
2881 AAATTTTAAC GGACTCTAAA TATTAAATGT GTTTTTATCT AGATATTAAA ACTTATAAAT
2941 AAGTGATATA TATTTTTAAT TTTAAGTAAC GTATTTATAT TTATGTGTCA TTTGTTTATT
3001 CTTTATGGGA TATATATGTG GTCTGAGGTA TATTTACTCA TCCATTGATT CAATGGTAAT
3061 TTACTGATTC AGTGTATCAC TGTTGATTTT ACGCATGAAG TGTTGTTCAA CACATGCCTC
3121 TTTCTAAATC TCGACAAAAT CAAAATTAGC AGACTCTGTC CAAATGGAAT AAGTAAACCA
3181 TCACTGTTGG TTCTTCTTTT TTTTTTTTTT TTTTTTTTTT TTTTGGTCGA GAAACTTTTC
3241 AAAAAGGTGC ATTTCTTTTG CAATCGGAGT TTTGTGAATT TCTACAAGAG AAGAATATGG
3301 CCAACTTTTT ACACTGACTG GATGCTAAAA TTCAAAGTTG TAGGTGTCAG TCAACCCACC
3361 TAACGTGGCA GCCCCGTTTT CTTTTAATAT GTTCACATTT ATATCCTCTA ACTCAAGATG
3421 CTTTTTATTT TATTTTAATT GTTTTTGTTT TATTTACTGT AGCTTTCAAA ATTGTTAAAT
3481 ATTACATAAG TAGTTTGTGT TCGGGTGTCA ACATAAAATA GTCCATCAAT TTGTTTTTTG
3541 TCTCAGCTTC ACTACCTAAT TAGCTATATA AAATGAAATT AATAGATGAA CTGAAATAAA
3601 TAAATAACAT ATATATATAA TATTTTTTTA AAAATTTAAG TGATTAAATA GATGTTATTC
3661 ATTATTTTTG GTGCACAGAT AGAACCGATA TTTAAACTAA TCAAACAATT TCTTTATCTT
3721 CTTCACATTT TTTTTGAGAA TTATCAAAAG TAGATTTAGA TTAAATCTAA AGTCTCCAAC
3781 TACAATCCCA AATTAGAGAT TTTTTTTTTT CAAAACAAAG CTGAATAGTA CTGTGAAAAG
3841 GGTAGCAAAG TAGGCAAGTA ATAGCAGTCT ATTTGGTAAT GACAGTTTTT GTAATAAAAA
3901 CAAGAGAGAT GGGTTGTGAG TACACCTGGA TGTTTGCATG AACTGCTACT TGATATATAG
3961 TGCCTGGATT GCGCCAGGTC TACAAAAGCA AATTTCTCTT TTCTCCTTTT TATTTTATTT
4021 ATTTATTTAT TTTTTCTCTC TATTCTATTA CTTGTTTGTT GGTCAGATCC CAGTAACAGA
4081 TTTGGCCTCA CTTTTCTCTG TAATCACTAA TTAGTATTCA GTGTGGAAGT CACGTGACGC
4141 CTCTTTTACT AAGACAATAA AAGAAAAGCT CACGTTCTTC TCAACAACAA GAACAGAAAT
4201 AAAAGCTTTT TCTCACATTT GCCAAAAGCC CCAAATCTCA GATCTTACAC TTTTCCCTTC
4261 ATATATTTTT TATCAACACT GTCATATTAT GTTTGTACGA TCCTTTTTTC TTTATTTTTT
4321 TTAAGAAAAA AAAGTTACAA ACATTTTCTT TTTCCACAAT AGTTTATTTA GGGGTTACAT
4381 ACTTTCCAAA TACAAATATA TTTTTAAAAG ATAATAATAG TTTCATTTTA GTCATAAAAT
4441 TTTAATTTAT ATCATCCAAT TTTAATCTTA TTTCAATTTA GTTATTTTGT AAATAAAAAA
4501 TTGACACAGA CAAGAATAAC TAACAAGAAA AGAGTAATTG AGGAAGAAAA ATATGACATG
4561 TCAATTTTTT ATTGGCAAAG TGACTAAATT GCAACTAAAA CTAAAGTTAA ATGATTAAAA
4621 TGTAACAAAT AAAAATTTTG TAACCAAAAT GAAACTAAGT GCAAAGTTAA GTGACAATTA
4681 ACTATATTTT TATTAGAAAG ATCTTGTTTA CACGGGATGT TTATTTTCAC ATATATACCA
4741 AATTTGATAA ATAAAAATAC ATTTAATTTT AAGTATATGT ATCGAAGTGT AAAAAATTTA
4801 TATATATATA CACACACAAC TCGTAAAATT CATTTATTCA TTACTCATTG TAATTACCCT
4861 ATTTGTTAAG AGATATTTTT TTGGTTTTAT CATGGTGTTT TGGTCATTTC GACCAAAACA
4921 CTATTTTTAT GGATTTTGGG AGCAGTTGTA TATTTTTACC ACATTCAGCC TCTACTTAGT
4981 AAACAAAATG TTATCTAATT TTACTAGGGA AGAACTGTGA GAACTAACTT GGTTGGACTG
5041 GGTTGTTAAT AATTTTATAT TCTACCATAA AGAGACTTAA AGCACTCATT ATTAGCCACA
5101 TAGAGTATAT ATTTTGATCT ATTAAGCTGC ACTGCGGAGG CTACTTTTTA AATATTAACT
5161 TCTAAAAAAA ATTAATTTTA GTTAATCAAG ATTTATATTT CTTAAATGTT ATTC**AG**ACAC

5221 ACAAGAGGAG GCAGCAGCAG CATATGATAT GGCAGCAATT GAGTACAGAG GAGCAAATGC
5281 TGTGACCAAT TTTGATATCA GCAATTACAT AGACCGCTTG AAGAAGAAAG GCATCCTTTT
5341 AGACCAGATA CTCCCTGATC AACCACTTAG AAAATGCTCT AGCGAATCTG AAGAAGCAGA
5401 AGCAGAAGCA GAAGTTGAAC GACTGCCGTC ACTGCCCTCA TCGATATTAC CACAAGAACA
5461 GGACACAATC TCACCACAAC TTCAATGCAC ACAGCTCCTT CCATCCATGG ACTCTTGCAC
5521 AATGATTAAT ATGGACCCTA TCGAAGATAA TGAGCTCACC TGGAGTTTTT GCTTGGATTC
5581 TGGGCTGACC CTTTTCTCAG TACCCGAGCT CCCCCTCGAA AATGCTTGCG AGCTACCGGA
5641 CTTGTTCGAT GACACAGGCT TTGAAGACAA TATAGACTTG ATATTTGATG GGTGTTGCTT
5701 TGGAAATGAT GATGACGGTG GTGGTGGTGC CAACCACCAG GAATTTATGG TGGAAAGTAG
5761 AGGATGTAGG GTAGGGGAGG TTGGGATTTC AGGGAGCATG GAGGAGGAAA ACGGGAAGGA
5821 GATGTGTTGC AGTTCAAGTT CGCCATCTTG TTCAACAACC ACCTCGGTTT CTTGTTGTAA
5881 CTATTCTGTT TGAATTGCCG TTTTCTTTGG TTCTGGTTTT AGCTTTGGTC GAGGTTTGTT
5941 TGTTTGTTTG TGGGTAGATA TAATATCACA TGGGAAATCA GAGGATGGGG AAAATGTACT
6001 TTTTTTCGAA AGTAATTGGT GTGTTGATCG CCCCGATATG CATATCCGGA ACCTCATCTA
6061 TTTCCAATAA CAAAGTTATT ATAGTTGATA GAATAAATAA AACTTCTAGG CTCCTTTTAA
6121 ATTTTTTGAA AGATTGGTGT TTCCCCTATA GTTATAACAT ATATTGCAAA AGTGCATTAG
6181 ACATATTTCT GTTAATCTAA AGTTTTAAAT AAAATCTAAT TAAATGTGTA AAGATTTTTA
6241 AAATAGTATT TTGTCGCTTG TAAAGTTTTG TCTGTCATGC TCTATATTAA TTTCGAACCG
6301 AACATATAAA CATTAAAAAA ATATAATTAA ATGCGTTAAA AAATTATAAT TAAGATAAAT
6361 TACATAAAAC TTTACAATTT ATATTAAAAA AATCATTTTC TTATTATATT TTTTTATTAC
6421 AAACTAAATT GTTATTAAAT TTTAATTTTA ATAACACACC AATAATTTTT TTACAGCTAA
6481 TGTGTAGGTT TGAATGAATA ATATTAGATT TTGCATTTTG TAATAAAAAA GATATCAATT
6541 TTTATTATTT TTATAACTAA ATCATTATTA AATTTTAATT TTAATAACAC CAATTGTTCT
6601 ATAGCAAATG AGATACATAA TCCTTATTAT GCACTTTAAA ATTAATTTAC TCTCTTCTCT
6661 CTTCTTCTTA GACAAAGTTA CAAATTCTTT CCTACAAATA TGGTAAAATT AAATAAATAG
6721 AAAAAGAAGA GAGTATATTA GTTTTAGGAT TTTAGGCTAA TTCTTATGTA TCCAATTGCT
6781 TACTAAATTT AATGATACAA GTTATTTCTT CCATGTGTCA ATGATGATGT GGGGTTAGAG
6841 AATGAATAGT TTTTAAATAT TTATTTTCAA ATGGTACATC ATCCAAGTAT ATCAAAAAAA
6901 TTCACTGGTA TTATTAAAAT TAAA

Figure S1. RcWRI1 genomic sequence.The exonis labeled in red, and the intron is indicated in blue. The intron boundary signals (AG and GT) are indicated in bold blue letters.
