# Supplementary material for: Splice Variants of the Castor WRI1 Gene Upregulate Fatty Acid and Oil Biosynthesis When Expressed in Tobacco Leaves
Source: Int J Mol Sci. 2018 Jan 5;19(1):146. doi: 10.3390/ijms19010146 (PMC5796095; doi:10.3390/ijms19010146)
Supplement: Supplementary file 1 [file ijms-19-00146-s001.zip › Figure S2 .pdf]

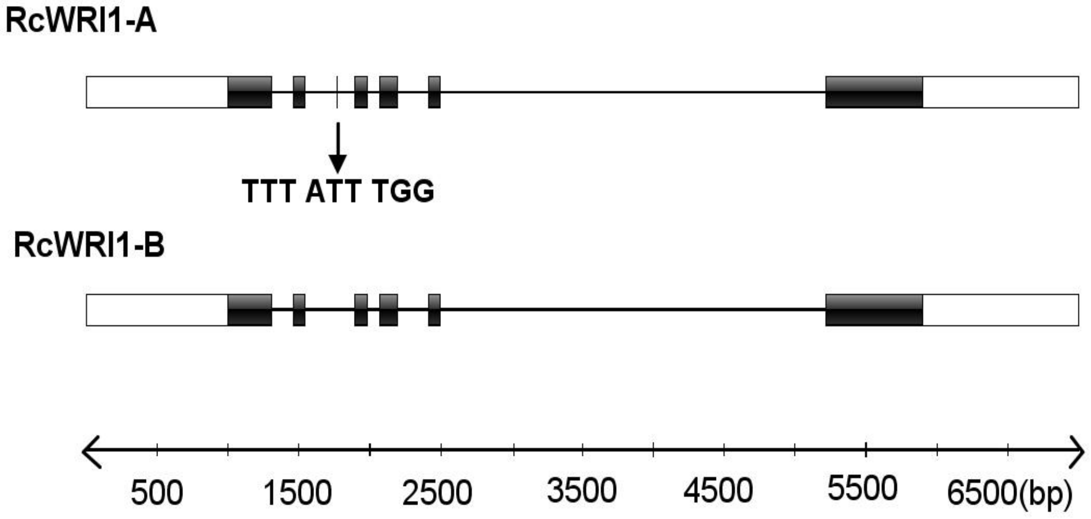

Figure S2. The gene structureof splice formsRcWRI1-A and RcWRI1-B. The splice form A (RcWRI1-A) contains 9-bp (TTTATTTGG) exon encoding “VYL”, which is absent in the splice form B (RcWRI1-B).
