# Supplementary material for: Splice Variants of the Castor WRI1 Gene Upregulate Fatty Acid and Oil Biosynthesis When Expressed in Tobacco Leaves
Source: Int J Mol Sci. 2018 Jan 5;19(1):146. doi: 10.3390/ijms19010146 (PMC5796095; doi:10.3390/ijms19010146)
Supplement: Supplementary file 1 [file ijms-19-00146-s001.zip › Figure S3 .pdf]

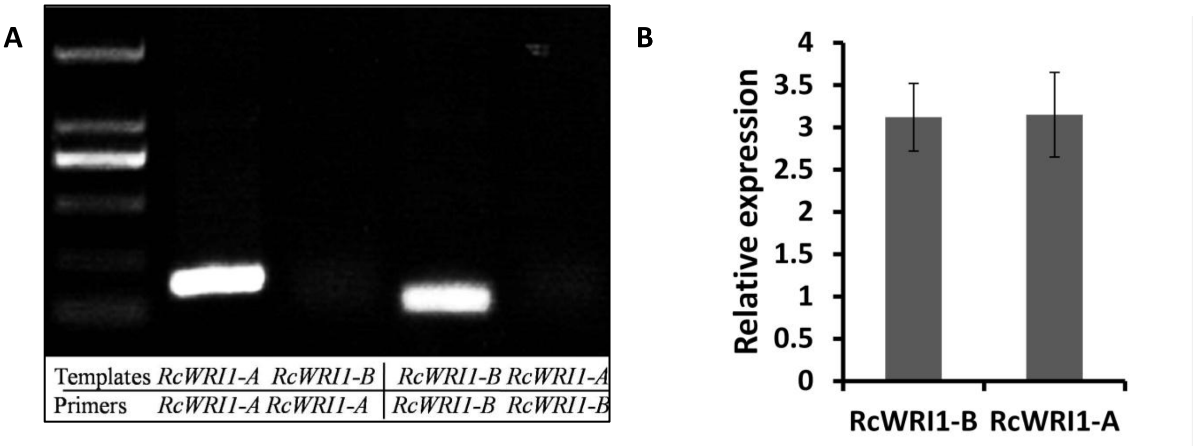

Figure S3. A. Verification of the primer specificity of two RcWRI1 isoforms. Plasmids of were used as templates in PCR analysis. Specific PCR products were analyzed on 1% agarose gels. B.The relative expression of *RcWRI1-A* and *RcWRI1-B* in tobacco leaves either expressing *RcWRI1-A* or *RcWRI1-B*. Total RNAs were isolated from the leaves and used for qRT-PCR analysis. Each value is the mean ± SD of six biological replicates.
